# A New Validity Index Based on Fuzzy Energy and Fuzzy Entropy Measures in Fuzzy Clustering Problems

**DOI:** 10.3390/e22111200

**Published:** 2020-10-23

**Authors:** Ferdinando Di Martino, Salvatore Sessa

**Affiliations:** 1 Dipartimento di Architettura, Università degli Studi di Napoli Federico II, Via Toledo 402, 80134 Napoli, Italy; sessa@unina.it; 2 Centro Interdipartimentale di Ricerca “Alberto Calza Bini”, Università degli Studi di Napoli Federico II, Via Toledo 402, 80134 Napoli, Italy

**Keywords:** FCM, validity index, fuzzy energy, fuzzy entropy

## Abstract

Two well-known drawbacks in fuzzy clustering are the requirement of assigning in advance the number of clusters and random initialization of cluster centers. The quality of the final fuzzy clusters depends heavily on the initial choice of the number of clusters and the initialization of the clusters, then, it is necessary to apply a validity index to measure the compactness and the separability of the final clusters and run the clustering algorithm several times. We propose a new fuzzy C-means algorithm in which a validity index based on the concepts of maximum fuzzy energy and minimum fuzzy entropy is applied to initialize the cluster centers and to find the optimal number of clusters and initial cluster centers in order to obtain a good clustering quality, without increasing time consumption. We test our algorithm on UCI (University of California at Irvine) machine learning classification datasets comparing the results with the ones obtained by using well-known validity indices and variations of fuzzy C-means by using optimization algorithms in the initialization phase. The comparison results show that our algorithm represents an optimal trade-off between the quality of clustering and the time consumption.

## 1. Introduction

A validity index is a measure applied in fuzzy clustering to evaluate the compactness of clusters and the separability among clusters.

Numerous validity indices have been applied to measure the compactness and separateness of clusters detected by applying the fuzzy C-means (FCM) algorithm [1,2].

The two well-known main drawbacks of the FCM are the random setting of the initial clusters and the requirement of assigning the number of clusters in advance. The initial selection of the cluster centers can affect the performances of the algorithm in terms of efficiency and number of iterations needed to obtain the convergence. Moreover, the quality of the final fuzzy clusters depends on the choice of the number of clusters, then, it is necessary to use a validity index to evaluate what is the optimal number of clusters.

A simple technique applied to solve these problems is to execute the clustering algorithm several times, varying the initial centers of the clusters and the number of clusters, and to choose the optimal clustering using a validity index to measure the quality of the final clustering. However, this technique can be computationally expensive as the clustering algorithm has to be run many times.

In References [3,4], a technique is proposed which is based on the subtractive clustering algorithm to initialize the clusters, but this method needs to set the maximum peak and the maximum radius parameters.

In Reference [5], a technique, called *Fuzzy Silhouette,* is proposed: this method generalizes the Average Silhouette Width Criterion [6] applied for evaluating the quality of crisp clustering. The authors of Reference [5] show that the proposed validity measure, unlike other well-known validity measures, such as Fuzzy Hypervolume and Average Partition Density [7] and the Xie-Beni [8] index, can be used as an objective function of an evolutionary algorithm to automatically find the number of clusters; however, this approach requires running FCM many times for each cluster number selection. 

In Reference [9], a new optimization method based on the density of the grid cells is proposed to find the optimal initial cluster centers and number of clusters: this approach can reduce run times in high-dimensional clustering. 

The K-means algorithm is used in Reference [10] to initialize the centers of the clusters; then, the Partition Coefficient [1,11] and Partition Entropy [12] validity measures are calculated to find the optimal number of clusters. The drawback of this method is that it is highly time consuming and it can be unsuitable for managing massive datasets.

Some authors propose hybrid FCM variations in which meta-heuristic approaches are applied to optimize the initialization of the cluster centers. In Reference [13], a kernel FCM algorithm is proposed in which an evolutive method is applied in order to find the initial cluster centers. A Genetic Algorithm (GA) is proposed in Reference [14] to find the optimal initial FCM cluster centers in image segmentation problems. A Particle Swarm Optimization (PSO) algorithm is proposed in Reference [15] to find the optimal FCM initial cluster centers for sentiment clustering. Three hybrid FCM algorithms, based on Differential Evolution, GA, and PSO methods, are proposed in Reference [16] to optimize the cluster centers’ initialization. These algorithms, while guaranteeing a higher quality of results, require too long execution times, and they too are unsuitable for handling high-dimensional data.

In this paper, we propose a FCM variation in which a new validity index based on the De Luca and Termini Fuzzy Entropy and Fuzzy Energy concepts [17,18] is used to optimize the initialization of the clusters and to find the optimal number of clusters. Our aim is to reach a trade-off between the time consumption and the quality of the clustering algorithm.

Recently, a weighted FCM variation based on the De Luca and Termini Fuzzy Entropy was proposed in order to optimize the initialization of the cluster centers in Reference [19]. To initialize the cluster centers, the authors initially execute a weighted FCM algorithm, in which the weight assigned to a data point is given by a fuzziness measure obtained by calculating the mean fuzzy entropy of the data point and then the initial cluster centers are found when the mean fuzzy entropy of the clustering converges as well.

The algorithm proposed in Reference [19] is less time-consuming than hybrid algorithms using meta-heuristic approaches, but like the algorithm proposed in Reference [10], it applies an iterative method of pre-processing to initialize cluster centers. Furthermore, it does not detect the optimal number of clusters that must be set in advance.

In the proposed algorithm, the validity measure of the quality of clustering based on the fuzzy energy and fuzzy entropy is calculated both in the pre-processing phase to find the optimal initial cluster centers and to determine the optimal number of clusters. We set the number of clusters and randomly assign cluster centers several times, by choosing as initial cluster centers those for which the clustering validity index is greatest; finally, the FCM algorithm runs. We repeat this process by increasing the number of clusters up to a maximum number. After obtaining the final clusters for each setting of the number of clusters, we choose the one with the largest validity index.

In Section 2, we give a brief review on the Fuzzy Energy and Fuzzy Entropy measures of a fuzzy set and of the FCM algorithm. In Section 3, we introduce the proposed FCM algorithm based on the fuzzy energy and entropy-based validity index. In Section 4, we present several experimental results to demonstrate the features of the proposed index by applying to FCM. In Section 5, we present our conclusions.

## 2. Preliminaries 

### 2.1. Fuzzy Energy and Entropy Measures

Let X be a universe of discourse and F(X) = {A: X → [0, 1]} be the set of all fuzzy sets defined on X. Moreover, let A ∊ F(X) and B ∊ F(Y) be two fuzzy sets defined on the sets X and Y respectively, and let R ⊆ F[X × Y] be a fuzzy relation on X × Y.

In References [17,18], two categories of fuzziness measures of fuzzy sets are defined: fuzzy energy and fuzzy entropy. If X = {x_1_, …, x_m_} is a discrete set with cardinality m, the energy measure of fuzziness of the fuzzy set A ∊ F(X) is given by:

(1) $$E(A)=\sum_{i=1}^{m}e\left(A\left(x_{i}\right)\right)$$

where e: [0, 1] → [0, 1] is a continuous function called fuzzy energy function. The following restrictions are required for the function e:

(1) e(0) = 0
(2) e(1) = 1
(3) e is monotonically increasing.

The simplest fuzzy energy function is given by the identity e(u) = u with u ∊ [0.1]. A more general formula for e(u) is:

(2) $$e\left(u\right)=u^{p}$$

where *p* > 0 is a positive number. 

The minimal value of the fuzzy energy measure is 0 and the maximal value is given by E(A) = Card(X) = m, where Card(X) is the cardinality of the set X.

The energy measures of a fuzzy set A can be seen as a measure of information contained in this fuzzy set. If E(A) = 0, then A coincides with the empty set; if E(A) = m, then A coincides with the set X.

The entropy measure of fuzziness of the fuzzy set A is given by:

(3) $$H\left(A\right)=\overset{m}{\underset{i=1}{\sum }}h\left(A\left(x_{i}\right)\right)$$

where h: [0, 1] → [0, 1] is a continuous function called fuzzy entropy function. The following restrictions are required for the function h:

(4) h(1) = 0
(5) h(u) = h(1 − u)
(6) h is monotonically increasing in [0, ½]
(7) h is monotonically decreasing in [½, 1].

The simplest fuzzy entropy function is given by:

(4) $$h\left(u\right)=\{\begin{array}{cc} 2u & if\;u\;<\;\frac{1}{2}\\ 2\left(1-u\right) & if\;u\;\geq \frac{1}{2}\; \end{array}$$

This fuzzy entropy function has a minimal value of 0 when u is 0 or 1 and a maximum value 1 when u = ½.

De Luca and Termini in Reference [18] propose the following fuzzy entropy function:

(5) $$h\left(u\right)=\{\begin{array}{cc} 0 & if\;u\;=0\\ -u\cdot log_{2}\left(u\right)-\left(1-u\right)\cdot {log}_{2}(1-u) & if\;\;0\;<\;u\;<1\\ 0 & if\;u\;=1\; \end{array}$$

This fuzzy entropy function has a maximum value 1 when u = ½, and it is called Shannon’s function.

The entropy measures of a fuzzy set A can be seen as a measure of the fuzziness contained in this fuzzy set. If H(A) = 0, then for each element x_i_, i = 1, …, m, A(x_i_) = 0 or A(x_i_) = 1 and A coincides with a subset of the set X; if H(A) = m, then for each element x_i_, i = 1, …, m, A(x_i_) = ½ and the fuzziness of A is maximum.

A problem is to find the fuzzy set from a family of fuzzy sets of F(X) with the highest information content and the lowest fuzziness.

### 2.2. Fuzzy C-Means Algorithm

Let **X** = {**x**_1_, …, **x**_N_} ⊂ R^n^ be a set of N data points in the n-dimensional space R^n^, where **x**_j_* = (*x_j1_, …, x_jn_), and let **V** = {**v**_1_, …, **v**_C_} ⊂ R^n^ be the set of centers of the C clusters. Let **U** be the C × N partition matrix, where u_ij_ is the membership degree of the j*th* data point **x**_j_ to the i*th* cluster **v**_i_.

The FCM algorithm [1,2] is based on the minimization of the following objective function:

(6) $$J\left(U,V\right)=\overset{C}{\underset{i=1}{\sum }}\overset{N}{\underset{j=1}{\sum }}u_{ij}^{p}d_{ij}^{2}=\overset{C}{\underset{i=1}{\sum }}\overset{N}{\underset{j=1}{\sum }}u_{ij}^{p}{\left\|x_{j}-v_{i}\right\|}^{2}$$

where d_ij_ = $\left\|x_{j}-v_{i}\right\|$ is the Euclidean distance between the center v_i_ of the i*th* cluster and the j*th* object **x**_j_, p ∈ [1, +∝] is the fuzzifier parameter (a constant which affects the membership values and defines the degree of fuzziness of the partition). For m = 1, FCM become a hard C-means clustering; the more m tends towards +∝, the more the fuzziness level of the clusters grows.

By considering the following constraints:

(7) $$\overset{C}{\underset{i=1}{\sum }}u_{\mathrm{ij}\;}=1\;\forall \;j\in \left\{1,\ldots ,N\right\}$$

(8) $$0<\overset{N}{\underset{j=1}{\sum }}u_{\mathrm{ij}}<\;N\;\forall \;i\in \left\{1,\ldots ,C\right\}$$

and applying the Lagrange multipliers, we obtain the following solutions for (1):

(9) $$v_{i}=\frac{\sum_{j=1}^{N}u_{\mathrm{ij}}^{p}x_{j}}{\sum_{j=1}^{N}u_{\mathrm{ij}}^{p}}\;i\in \left\{1,\ldots ,C\right\}$$

and

(10) $$u_{ij}=\frac{1}{\sum_{k=1}^{c}{\left(\frac{d_{ij}}{d_{kj}}\right)}^{\frac{2}{m-1}}}\;i\in \left\{1,\ldots ,C\right\},\;j\in \left\{1,\ldots ,N\right\}$$

An iterative process is proposed in Reference [2] as follows: initially the membership degrees are assigned randomly; in each iteration, the cluster centers are calculated by (4), then the membership degree components are calculated by (5). The iterative process stops at the t*th* iteration when

(11) $$\left|U^{\left(t\right)}-U^{\left(t-1\right)}\right|\leq \varepsilon \;i\;=\;1,\ldots ,C;\;j\;=\;1,\ldots ,N$$

where ε > 0 is a parameter assigned a priori to stop the iteration process and

(12) $$\left|U^{\left(t\right)}-U^{\left(t-1\right)}\right|=\underset{\begin{array}{c} i=1,\ldots ,C\\ j=1,\ldots ,N \end{array}}{\max}\left\{\left|u_{ij}^{\left(t\right)}-u_{ij}^{\left(t-1\right)}\right|\right\}\;i\;=\;1,\ldots ,C;\;j\;=\;1,\ldots ,N$$

The pseudocodes of the FCM algorithm (Algorithm 1) are shown below.
**Algorithm 1:***FCM***Input:** Dataset ***X*** = {**x**_1_, …, **x**_N_} 
**Output:** Cluster centers **V** = {**v**_1_, …, **v**_C_}; Partition matrix **U**

**Arguments:** number of clusters C; fuzzifier p; stop iteration threshold ε *Set* p, ε, C to the values of the arguments *Initialize* randomly the partition matrix **U** **Repeat**   *Calculate* **v**_i_ i = 1, …, C by using (9)   *Calculate* u_ij_ i = 1, …, C j = 1, …, N by using (10)**Until**$\left|U^{\left(t\right)}-U^{\left(t-1\right)}\right|>\varepsilon$**Return V,U**

## 3. The Proposed FCM Algorithm Based on a Fuzzy Energy and Entropy Validity Index

Let **X** = {**x**_1_, …, **x**_N_} be the set of data points with cardinality N. We consider the fuzzy set A_i_ ∊ F(**X**), where A_i_(**x**_j_) = u_ij_ is the membership degree of the j*th* data point to the i*th* cluster.

We propose a new validity index based on the fuzzy energy and fuzzy entropy measures to evaluate the compactness of clusters and the separability among clusters.

By using (1) and (3) respectively, we can evaluate the fuzzy energy and the fuzzy entropy of the i*th* cluster, measuring the fuzzy entropy and the fuzzy energy of the fuzzy set A_i_, given by

(13) $$E\left(A_{i}\right)=\frac{1}{N}\sum_{j=1}^{N}e\left(A\left(x_{j}\right)\right)=\frac{1}{N}\sum_{j=1}^{N}e\left(u_{ij}\right)\;i\;=\;1,2,\ldots ,C$$

(14) $$H\left(A_{i}\right)=\frac{1}{N}\sum_{j=1}^{N}h\left(A\left(x_{j}\right)\right)=\frac{1}{N}\sum_{j=1}^{N}h\left(u_{ij}\right)\;i\;=\;1,2,\ldots ,C$$

where the fuzzy energy and entropy are normalized dividing them by the cardinality N of the dataset.

Fuzzy energy (13) measures the quantity of information contained in the i*th* cluster and fuzzy entropy (14) measures the fuzziness of the i*th* cluster, namely the quality of the information contained therein.

For example, a cluster with low fuzzy entropy has low fuzziness, so it is compact; however, if it also has a low fuzzy energy, then the information which it contains is low. Hence, even if compact, a very small number of data points will belong to this cluster and this could be due to the presence of noise or outliers in the data. Moreover, a cluster with a high value of fuzzy entropy has high fuzziness and low compactness. 

We set the function (2) as fuzzy energy function, where p is given by the value of the fuzzifier parameter. The fuzzy entropy function h(u) is given by the Shannon function (5).

We measure the energy and the entropy of the clustering given by the averages of the energy and entropy of the C clusters:

(15) $$E=\frac{1}{C}\sum_{i=1}^{C}E\left(A_{i}\right)$$

and

(16) $$H=\frac{1}{C}\sum_{i=1}^{C}H\left(A_{i}\right),$$

respectively. The proposed validity index, called Partition Energy-Entropy (PEH), is given by the difference between the energy and the entropy of the clustering:

(17) PEH=E−H

This index varies in the range [−1, 1], the optimal clustering is the one that maximizes PEH, and the greater the value of PEH, the more the clusters are compact and well separated from each other.

We propose a new algorithm, called PEHFCM, in which the PEH index is used to initialize the cluster centers and to find the optimal number of clusters. 

In addition to the fuzzifier and iteration error threshold parameters, further arguments of the algorithm are the maximum number of clusters, Cmax, and the number of random selections of initial C clusters, Smax. The PEHFCM algorithm is composed of a For loop in which the number of clusters is initially set to 2 and then cyclically iterated until the Cmax value is reached. In each cycle, Smax sets of cluster centers are initially selected, for each of which the PEH index is calculated. The optimal set of initial cluster centers is the one for which the PEH indicator is maximum. Subsequently, a variation of the FCM algorithm is performed, called FCMV, which, unlike FCM, uses the set of initial cluster centers V^0^ as a further argument instead of setting it randomly. Finally, the PEH index of the final clustering is calculated.

The PEHFCM algorithm returns the optimal number of C* clusters and the respective sets of cluster centers V* and partition matrix U* corresponding to the highest PEH validity index.

Below, we show the algorithm PEHFCM (Algorithm 2) and the algorithm FCMV (Algorithm 3), called PEHFC.
**Algorithm 2:***PEHFCM.***Input:** Dataset ***X*** = {**x**_1_, …, **x**_N_} **Output:** Cluster centers **V** = {**v**_1_, …, **v**_C_}; Partition matrix **U**, optimal number of clusters C* **Arguments:** max num of clusters, Cmax; max num of random selections of the initial cluster centers, Smax, fuzzifier p; stop iteration threshold ε *Set* p, ε, Cmax to the values of the arguments C*:= 1PEH*:= –1 **For** c = 2 **to** Cmax**For** k = 1 **to** SmaxSet randomly the partition matrix **U**Calculate the value of the cluster centers **v**_i_ by (9) i = 1, …, c*Calculate* E by (15) Calculate H by (16)PEH: = E − H**If** PEH > PEH* **Then****V**^0^:= **V**_k_**End if**PEH*:= −1  *Call* FCMV(***X***, **V**^0^, p, ε, C)  *Calculate* E by (15)Calculate H by (16)PEH: = E – H**If** PEH > PEH* **Then**PEH*: PEHC*: cV*: **V**U*: = **U****End if****Next** cReturnU*, V*, C*



**Algorithm 3:**
*FCMV*
**Input:** Dataset ***X*** = {**x**_1_, …, **x**_N_} Initial cluster centers **V**^0^ = {**v**_1_^0^, …, **v**_C_^0^} **Output:** Cluster centers **V** = {**v**_1_, …, **v**_C_}; Partition matrix **U**
**Arguments:** Initial cluster centers **V**^0^ = {**v**_1_^0^, …, **v**_C_^0^}; number of clusters C; fuzzifier p; stop iteration threshold ε *Set* p, ε, C to the values of the arguments**v_i_**:= **v_i_**^0^ i = 1, …, C *Calculate* u_ij_ i = 1,…,C j = 1, …, N by using (10) **Repeat**   *Calculate* **v_i_**:= i = 1, …, C by using (9)   *Calculate* u_ij_ i = 1, …, C j = 1, …, N by using (10)**Until**$\left|U^{\left(t\right)}-U^{\left(t-1\right)}\right|>\varepsilon$**Return V,U**

We can evaluate the computational complexity of PEHFCM, considering that the computational complexity of the FCM algorithm is by O(N·n·c^2^·I), where N is the number of objects, n their dimension, c the number of clusters, and I is the number of iterations.

In PEHFCM, for not high Smax values, it is possible to neglect the complexity of the computation of energy and entropy measures of the initial Smax cluster centers, approximating the computational complexity by O(N·n·c^2^·I·Cmax), where Cmax is the maximum number of clusters and I is the mean number of iterations of each FCM execution. 

Then, PEHFCM has the same computational complexity of the FCM in which the measurement of a validity index is performed to calculate the optimal number of clusters.

Moreover, due to the problem of initialization of cluster centers, FCM is generally performed several times, increasing its computational complexity; on the other hand, PEHFCM does not need to be executed several times as the algorithm determines the initial centers of the optimal clusters.

To measure the performances of the proposed algorithm, we compare the results with the ones obtained by applying FCM and applying our method by using other well-known validity indices: Partition Coefficient (PC) [1,11], Partition Entropy (PE) [12], Fukuyama and Sugeno (FS) [20], Xie-Beni (XB) [8], and Partition Coefficient And Exponential Separation (PCAES) [21], described below.

The PC validity index is given by the formula:

(18) $$PC\left(C\right)=\frac{1}{N}\overset{C}{\underset{i=1}{\sum }}\overset{N}{\underset{j=1}{\sum }}u_{ij}^{2}\;\frac{1}{C}\leq PC\left(C\right)\leq 1$$

It measures the crispness of the clusters. The value C* is obtained when PC is maximum. 

The PE validity index is given by:

(19) $$PE\left(C\right)=\frac{1}{N}\overset{C}{\underset{i=1}{\sum }}\overset{N}{\underset{j=1}{\sum }}u_{ij}\log_{2}u_{ij}\;0\leq PE\left(C\right)\leq \log_{2}C$$

It measures the mean fuzziness of the clusters, and the optimal number of clusters, C*, is obtained when PE is minimum. 

The FS validity index is given by:

(20) $$FS\left(C\right)=\overset{C}{\underset{i=1}{\sum }}\overset{N}{\underset{j=1}{\sum }}u_{ij}^{m}{\left\|x_{j}-v_{i}\right\|}^{2}\;-\overset{C}{\underset{i=1}{\sum }}\overset{N}{\underset{j=1}{\sum }}u_{ij}^{m}{\left\|x_{j}-\bar{v}\right\|}^{2}$$

where $\bar{v}$ is the average of the cluster centers. The first term in (20) measures the compactness of the clusters, the other one the separability among the same clusters. The optimal number of clusters, C*, is obtained when FS is maximum.

The XB validity index is given by the formula:

(21) $$XB\left(C\right)=\frac{\sum_{i=1}^{C}\sum_{j=1}^{N}u_{ij}^{m}{\left\|x_{j}-v_{i}\right\|}^{2}}{N\cdot \underset{\begin{array}{l} i=1,\ldots ,C\\ j=1,..,N \end{array}}{min}\left({\left\|x_{j}-v_{i}\right\|}^{2}\right)}\;-$$

The numerator measures the compactness of the clusters, and the denominator indicates the separability between clusters. The optimal number of clusters, C*, is obtained when XB assumes the minimum value. 

The PCAES validity index is given by the formula:

(22) $$PCAES\left(C\right)=\frac{\sum_{i=1}^{C}\sum_{j=1}^{N}{u_{ij}}^{2}}{\underset{i=1,\ldots ,C}{min}\left(\sum_{j=1}^{N}{u_{ij}}^{2}\right)}-\overset{c}{\underset{i=1}{\sum }}exp\left(\frac{-\underset{\begin{array}{l} k=1,\ldots ,C\\ k\neq i \end{array}}{min}(\left\{{\left\|v_{i}-v_{k}\right\|}^{2}\right\}}{\frac{1}{C}\sum_{i=1}^{C}{\left\|v_{i}-\bar{v}\right\|}^{2}}\right)$$

where the vector $\bar{v}$ is the average of the cluster center. The first term in (22) measures the compactness of clusters, and the last term the separability among clusters. The optimal number of clusters, C*, is obtained when PCAES assumes the minimum value. 

We complete our comparisons by comparing our method with hybrid metaheuristic algorithms.

The comparison tests are performed on well-known UC Irvine (UCI) machine learning classification datasets (http://archive.ics.uci.edu/ml/datasets.html). We measure the quality of the results in terms of accuracy, precision, recall, and F1-score [22,23].

## 4. Results

We show the results obtained on a set of over 40 classification UCI machine learning datasets. In all experiments, we used an Intel core I5 3.2 GHz processor, m = 2, ε = 0.01, and Smax = 100.

**Figure 1:**
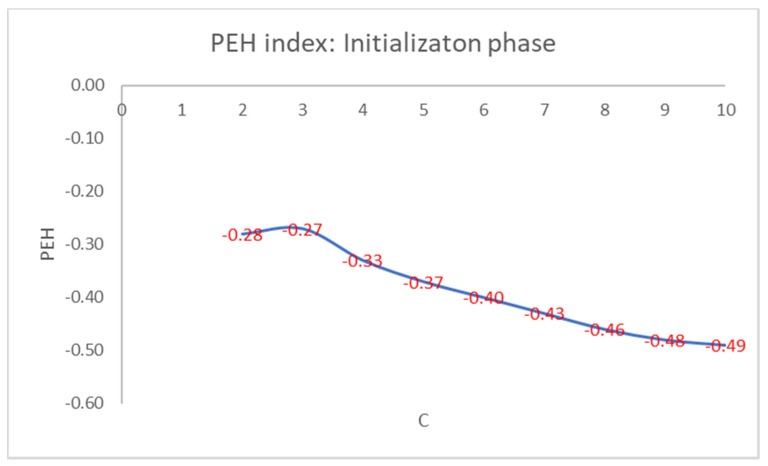
PEH index calculated in the initialization phase by varying the number of clusters.

For brevity, we only show in detail the results obtained on the well-known Iris flower dataset. This dataset contains 150 data points with 4 features given by the length and the width of the sepals and petals measured in centimeters: 50 data points are classified as belonging to the type of Iris flower Iris Setosa, 50 data points to the type Iris Versicolor, and 50 data points to the Iris Virginica type. Only the class Iris Setosa is linearly separable from the other two, which are not linearly separable. We set the max number of clusters, Cmax, to 10. In Figure 1, we show the values of the PEH index of the best initial cluster centers obtained for each setting of the number of clusters.

As can be seen from Figure 1, the maximum values of the PEH index are obtained for C = 3 by varying the number of clusters.

**Figure 2:**
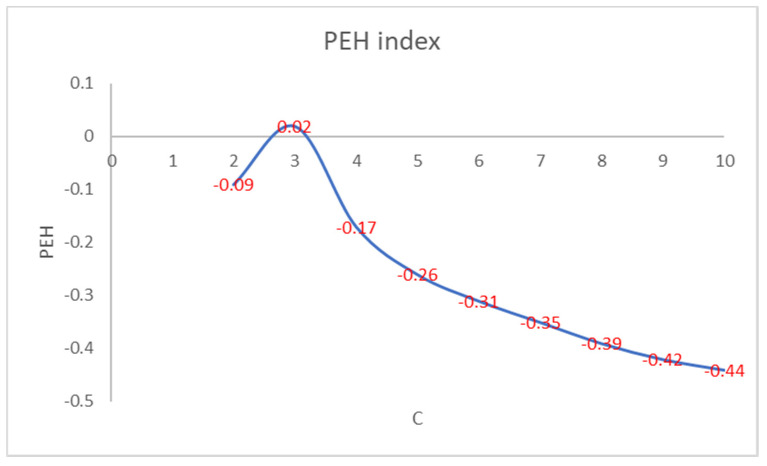
PEH index of the final clustering by varying the number of clusters.

**Figure 3:**
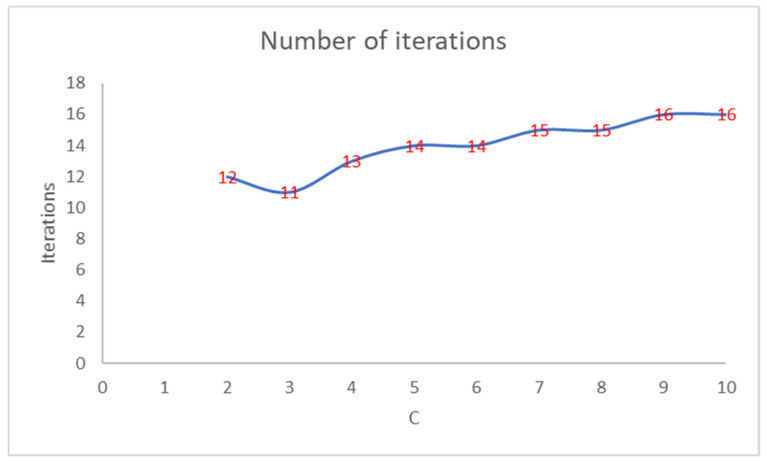
Number of iterations of the PEHFCM algorithm by varying the number of clusters.

Figure 2 shows that the number of iterations increases as the PEH value of the initial clustering decreases. Figure 3 shows the trend of the number of iterations necessary to reach the convergence by varying the number of clusters in PEHFCM. The least number of iterations (12) is obtained for C = 3.

**Figure 4:**
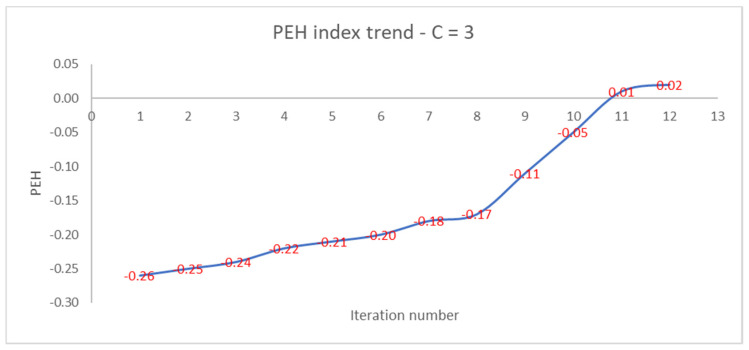
Trend of PEH with respect to the iteration number for C = 3.

Like the PEH index of the final clustering, the number of iterations increases as the PEH value of the initial clustering decreases. In Figure 4, we show the trend of the PEH in any iteration for C = 3.

The PEH index increases slightly, then increases rapidly after the 8th iteration and reaches a plateau at the 12th iteration. We compare the performances of the PEH index with the ones of the PC, PE, FS, and XB validity indices. 

Table 1 shows the optimal number of clusters found using the validity index, the number of iterations necessary for the convergence, and the running time. 

The best results are obtained by executing PEHFCM with respect to FCM + PC and FCM + PE (resp., FCM + FS and FCM + XB) when the optimal number of clusters obtained is 2 (resp., 3). In both cases, the least number of iterations and the shortest execution time are achieved using PEHFCM. In addition, we compare the results obtained by executing PEHFCM with the ones obtained via the entropy-based weighted FCM algorithm (EwFCM) [19] and the metaheuristic PSOFCM proposed in Reference [15].

Table 2 shows the running time, the accuracy, precision, recall, and F1-Score obtained by executing FCM + FS, FCM + XB, FCM + PCAES, PEHFCM, EwFCM, and PSOFCM.

The results in Table 2 show that the best classification performances are given by EwFCM and PSOFCM. PEHFCM has the shortest running time and classification performances comparable with EwFCM and PSOFCM.

These results are confirmed by testing other UCI machine datasets. Here, we present the results obtained on the Wine dataset. This dataset is given by 178 data points having 13 features: each data point represents an Italian wine derived from a specific crop and their features provide information on its chemical composition. The dataset is partitioned in three classes, corresponding to three crops.

In Table 3, we show the results obtained by considering the five validity indices.

Even in this case, PEHFCM provides the best number of iterations and running time.

Table 4 shows the running time and the classification performances of all the compared algorithms.

Also, here, the results obtained on the Wine dataset show that PEHFCM provides the shortest execution time and classification performances comparable to those obtained by using EwFCM and PSOFCM. 

In Table 5, the accuracy values obtained for some datasets used in our comparison tests are shown. These results confirm that the accuracy performances provided by PEHFCM are better than the ones provided by FCM + FS, FCM + XB, and FCM + PCAES, and are comparable to those provided by EwFCM and PSOFCM. 

We summarize the results obtained on all the classification UCI machine learning datasets used in our tests, calculating:

- The mean percent of gain (or loss) of running time. If T_C_ is a running time calculated by running a FCM-based method and T_CPEH_ is the one calculated with PEHFCM, this index is given by the average of the percentage of (T_CPEH_ − T_C_)/T_CPEH_. This value is equal to 0 for PEHFCM.
- The mean percentage gain (or loss) of a classification index. If I_C_ is a classification index value obtained by running a FCM-based method and I_CPEH_ is the one obtained with PEHFCM, this index is given by the average of the percentage of (I_C_ − I_CPEH_)/I_CPEH_. This value is equal to 0 for PEHFCM.

If the value of a summarized index is positive, then, by executing the algorithm, we obtain a gain in terms of running time or of the classification index; conversely, we get a loss if that value is negative. In Table 6, we show these results.

The results in Table 6 show that PEHFCM provides the best running time; indeed, the running times measured executing the other FCM-based algorithms were more than 28% longer than the one obtained by executing PEHFCM. The gain of accuracy, precision, recall, and F1-score obtained executing EwFCM and PSOFCM was less than 2%.

## 5. Conclusions

We proposed a variation of FCM in which a validity index was based on the fuzzy energy and fuzzy entropy of the clustering, in order to find an optimal initialization of the cluster centers and the optimal number of clusters.

The proposed method represents a trade-off between the running time and the clustering performances: it aims to overcome the problems of initializing cluster centers and setting the number of clusters a priori, without, at the same time, requiring long execution times due to the pre-processing phase necessary to optimize the initialization of cluster centers.

The results of experimental tests applied on well-known UCI machine learning classification datasets showed that the PEHFCM algorithm provides shorter running times than the EwFCM and PSOFCM algorithms, which use an optimization method based on fuzzy entropy and a metaheuristic PSO-based method to determine the initial cluster centers, respectively. Furthermore, PEHFCM provides classifier performance comparable to EwFCM and PSO FCM. 

Since the proposed method is FCM-based, its computational complexity depends on the number of data points and the number of features, and it may be unsuitable for managing massive and high-dimensional datasets. In the future, we intend to adapt PEHFCM to manage high-dimensional and massive datasets, in which it is essential to guarantee high performances both in terms of quality of results and execution times. 

## Figures and Tables

**Table 1:** Iris Dataset: validity indices comparison of number of iterations and running time.

Method	Number of Clusters	Iterations	Running Time (s)
**FCM + PC**	2	15	0.158
**FCM + PE**	2	15	0.157
**FCM + FS**	**3**	13	0.138
**FCM + XB**	**3**	13	0.135
**FCM + PCAES**	3	13	0.133
**PEHFCM**	**3**	**11**	**0.103**

**Table 2:** Dataset Iris: Comparisons of running time and classification performances.

Index	FCM + FS	FCM + XB	FCM + PCAES	PEHFCM	EwFCM	PSOFCM
**Running time (s)**	0.138	0.135	0.139	0.103	0.112	0.134
**Accuracy**	94.22%	94.67%	94.67%	96.00%	96.44%	96.44%
**Precision**	91.33%	92.00%	92.22%	94.00%	94.67%	94.67%
**Recall**	91.34%	92.00%	92.21%	94.01%	94.66%	94.67%
**F1 Score**	91.33%	92.00%	92.21%	94.00%	94.66%	94.67%

**Table 3:** Wine Dataset: validity indices comparison of number of iterations and running time.

Method	Number of Clusters	Iterations	Running Time (s)
**FCM + PC**	2	17	0.166
**FCM + PE**	2	17	0.168
**FCM + FS**	3	16	0.152
**FCM + XB**	3	16	0.151
**FCM + PCAES**	3	16	0.146
**PEHFCM**	3	12	0.116

**Table 4:** Wine Dataset: Comparisons of running time and classification performances.

Index	FCM + FS	FCM + XB	FCM + PCAES	PEHFCM	EwFCM	PSOFCM
**Running time (s)**	0.152	0.151	0.154	0.116	0.137	0.145
**Accuracy**	90.16%	90.34%	90.45%	92.91%	93.50%	93.81%
**Precision**	85.89%	86.03%	86.06%	88.67%	90.04%	90.61%
**Recall**	86.09%	86.18%	86.21%	88.61%	90.48%	90.84%
**F1 Score**	85.99%	86.10%	86.13%	88.64%	90.26%	90.72%

**Table 5:** Accuracy comparisons for some UCI machine learning datasets.

Dataset	Features	Data Points	Parameter	FCM + FS	FCM + XB	FCM + PCAES	PEHFCM	EwFCM	PSOFCM
**Breast cancer**	**32**	**569**	**Number of clusters** **Accuracy**	2	2	2	2	2	2
				84.41%	84.29%	84.37	94.75%	95.32%	95.68%
**Glass**	**9**	**214**	**Number of clusters** **Accuracy**	6	6	6	6	6	6
				88.70%	88.72%	88.78%	89.68%	89.45%	89.70%
**Iris**	**4**	**150**	**Number of clusters** **Accuracy**	3	3	3	3	3	3
				94.22%	94.67%	94.67%	96.00%	96.44%	96.44%
**Seeds**	**7**	**210**	**Number of clusters** **Accuracy**	3	3	3	3	3	3
				89.48%	89.42%	89.55%	90.64%	90.75%	90.81%
**Sonar**	**60**	**208**	**Number of clusters** **Accuracy**	2	2	2	2	2	2
				72.18%	72.27%	72.27%	78.41%	78.83%	78.76%
**Vehicle**	**19**	**846**	**Number of clusters** **Accuracy**	4	4	4	4	4	4
				63.21%	63.10%	63.15%	65.64%	65.90%	66.08%
**Wine**	**13**	**178**	**Number of clusters** **Accuracy**	3	3	3	3	3	3
				90.16%	90.34%	90.45%	92.91%	93.50%	93.81%

**Table 6:** Average percentage of gain of running time and classification indices.

Index	FCM + FS	FCM + XB	FCM + PCAES	PEHFCM	EwFCM	PSO FCM
Percentage gain of running time	−39.16%	−39.01%	−38.94%	0.00%	−28.06%	−31.57%
Percentage gain of accuracy	−3.14%	−3.11%	−3.06%	0.00%	+0.91%	+0.93%
Percentage gain of precision	−3.28%	−3.25%	−3.19%	0.00%	+1.74%	+1.77%
Percentage gain of recall	−3.22%	−3.26%	−3.18%	0.00%	+1.71%	+1.74%
Percentage gain of F1 Score	−3.25%	−3.25%	−3.19%	0.00%	+1.72%	+1.76%
